# In search of the origin of crown Mysticeti

**DOI:** 10.1080/03036758.2023.2249410

**Published:** 2023-08-24

**Authors:** Cheng-Hsiu Tsai

**Affiliations:** Department of Life Science, Institute of Ecology and Evolutionary Biology, and Museum of Zoology, National Taiwan University, Taipei, Taiwan

**Keywords:** Toothed mysticete, Baleen whale, Phylogeny, Oligocene, New Zealand

## Abstract

Recent research on mysticete fossils from the Late Eocene and Oligocene has revolutionised our understanding of the diversity and evolutionary scenarios for early baleen whales. For example, aetiocetids are a possible, though controversial, lineage that bridges the gap between the toothed and baleen-bearing mysticetes, and eomysticetids show a further transitional step towards the baleen-bearing status, with the presence of non-functional dentition in adults. However, information about the origin of crown mysticetes, including the most recent common ancestor of all extant lineages and its descendants, is critical to further understanding the evolution of baleen whales. The phylogenetic positions of the Oligocene *Toipahautea*, *Whakakai*, *Horopeta*, and *Mauicetus* from New Zealand remain unresolved and problematic, but all four genera show a close relationship with crown mysticetes. The original and subsequent cladistic analyses have consistently revealed a sister relationship between the *Toipahautea-*to*-Mauicetus* grade and crown mysticetes, and *Horopeta* has been placed close to the cetotheriids within the crown group. This review aims to stimulate more research on this topic by elucidating the origin of crown mysticetes, which likely experienced a poorly known radiation event during the Oligocene that established the modern lineages.

## Almost like a ‘baleen’ whale

Baleen whales (Mysticeti) include the blue whale (*Balaenoptera musculus*), the largest animal to have ever lived on Earth – and even the smallest extant baleen whale, the pygmy right whale (*Caperea marginata*), reaches more than 6 metres in length (Wilson and Mittermeier 2014; Würsig et al. 2018). Consequently, the evolution of body size in baleen whales has been assessed from various aspects (Tsai and Kohno 2016; Slater et al. 2017; Bianucci et al. 2019; Bisconti, Pellegrino, et al. 2021). Research findings to date indicate that the key driving factor for gigantism in these animals is likely related to their feeding strategy and efficiency (Goldbogen et al. 2019), i.e. the origin of their characteristic feature, baleen.

Studies on early development have shown that baleen whales possess functional dentition at the early ontogenetic stage, but the feeding apparatus (teeth) is subsequently replaced with keratinous baleen (Deméré et al. 2008; Lanzetti 2019). Interestingly, early discoveries of fossil whales with functional dentition were not associated with toothed ‘baleen’ whales but were instead recognised as archaeocetes (Pritchard 1939; Emlong 1966). However, further discoveries and detailed examinations resulted in the existence and recognition of toothed ‘baleen’ whales becoming well established (Valen 1968; Fordyce 1982; Barnes et al. 1994; Fitzgerald 2010). Furthermore, it has even been proposed that the toothed ‘baleen’ whales (Aetiocetidae) simultaneously possess proto-baleen and functional dentition (Deméré et al. 2008; Ekdale and Deméré 2022), showcasing the critical transition between toothed and baleen-bearing mysticetes (but also see alternative interpretations discussed by Ekdale and Deméré [2022]). Eomysticetids bridge the next gap between toothed (Aetiocetidae, Coronodonidae, Llanocetidae, and Mammalodontidae) and true (crown Mysticeti) baleen whales, both morphologically and phylogenetically. Additionally, eomysticetids represent a diverse early baleen whale lineage and consequently have attracted much research attention (Okazaki 2012; Boessenecker and Fordyce 2015a, 2015b, 2017a, 2017b; Hernández-Cisneros and Nava-Sanchez 2022) since the establishment of this family in 2002 (Sanders and Barnes 2002a).

In contrast to the burgeoning research on toothed mysticetes and eomysticetids, the origin and early evolutionary history of crown mysticetes have rarely been discussed. Crown mysticetes include the most recent common ancestor of all extant baleen whales (e.g. Balaenopteridae: blue whale, fin whale, and humpback whale; Balaenidae: right whales and bowhead whale) and its descendants. Toothed mysticetes (e.g. Aetiocetidae, Coronodonidae, Mammalodontidae, and Llanocetidae) are critical to deciphering the major transition between toothed and baleen-bearing whales. However, toothed mysticetes contribute little to interpreting the origin of crown mysticetes due to their distant phylogenetic relationship with other crown mysticetes. Eomysticetids consistently form a monophyletic clade outside crown mysticetes and exhibit primitive features, such as extremely elongated nasals, an anteroposteriorly elongated and narrow intertemporal region, and the presence of the elliptical foramen of the tympanic bulla, suggesting early divergence of this lineage. Examples of eomysticetid synapomorphies include parallel medial and lateral margins of the zygomatic process in dorsal view, the presence of a secondary squamosal fossa, and a sharp and well-developed ventromedial (or involucral) ridge on the bulla (see Boessenecker and Fordyce (2015b) for a detailed discussion on eomysticetid monophyly, and see Boessenecker et al. (2023) for the inclusion of *Sitsqwayk* and *Maiabalaena* in Eomysticetidae).

**Figure 1.**
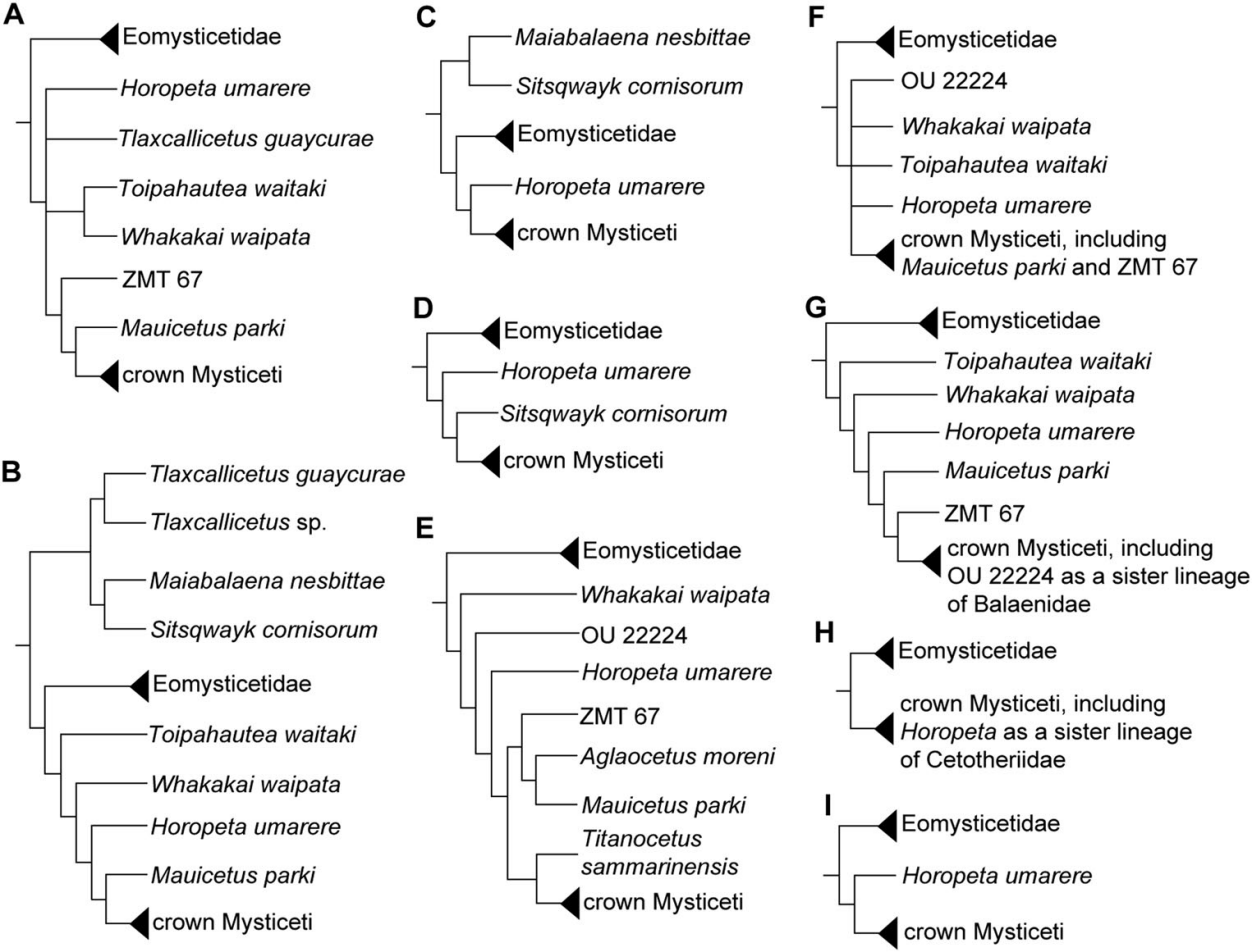
Simplified phylogenies showing the position of the *Toipahautea*-to-*Mauicetus* grade from the Oligocene deposits of New Zealand. **A**, Simplified phylogeny from Boessenecker et al. (2023); **B**, simplified phylogeny from Hernández-Cisneros and Nava-Sanchez (2022); **C**, simplified phylogeny from Shipps et al. (2019); **D**, simplified phylogeny from Bisconti, Damarco, et al. (2021); **E**, simplified phylogeny from Fordyce and Marx (2018); **F**, simplified equal-weighting phylogeny from Tsai and Fordyce (2018); **G**, simplified implied-weighting phylogeny from Tsai and Fordyce (2018); **H**, simplified equal-weighting phylogeny from Tsai and Fordyce (2015b); **I**, simplified implied-weighting phylogeny from Tsai and Fordyce (2015b).

By contrast, phylogenetic analyses have repeatedly placed some Oligocene mysticetes from New Zealand (e.g. *Toipahautea waitaki*, *Whakakai waipata*, *Horopeta umarere*, and *Mauicetus parki*) more crownward of eomysticetids and sister lineages of crown mysticetes, but not in a monophyletic lineage, forming a what is referred to here as a *Toipahautea*-to-*Mauicetus* grade. Of particular note, *Horopeta umarere* has been well nested in crown mysticetes, alongside the cetotheriids or the oldest cetotheriid (synapomorphies with other cetotheriids include a well-developed crista transversa of the periotic and a horizontally-oriented tympanic sulcus of the bulla), indicating a possible vital role in the evolution of early crown mysticetes (see Figure 1 for a compilation of various phylogenetic trees related to the position of *Toipahautea*, *Whakakai*, *Horopeta*, and *Mauicetus*).

This review aims to summarise progress to date in deciphering the origin and early evolution of crown mysticetes in the hope of stimulating further research on this topic. The development of keratinous baleen is a key morphological and evolutionary innovation that has greatly contributed to the success and gigantism of baleen-bearing mysticetes. This innovation also likely resulted in early adaptive radiation at the node of origin of crown mysticetes and may have caused high evolutionary rates, obscuring our ability to recognise early crown mysticetes that were ancestral to modern mysticete lineages.

## Stem-to-crown transition

Groups of extinct species that are basal to the most recent common ancestor of the study group on the phylogenetic tree are defined as stem lineages. Phylogenetically speaking, this definition is reasonable because phylogenetic reconstructions only suggest sister-group relationships. However, ancestor–descendant relationships are key to revealing the hidden transition or early evolutionary history of a group (Tsai and Fordyce 2015a). For example, archaeocetes (now commonly known as stem Cetacea) have long been considered a monophyletic lineage and recognised as a separate suborder, Archaeoceti (Flower 1883; Kellogg 1936), but it is now clear that this is a paraphyletic group (Uhen 2010). Furthermore, basilosaurids are most likely the lineage that gave rise to neocetes (or crown Cetacea), including mysticetes and odontocetes (Uhen 2010). Therefore, archaeocetes did not go extinct, but rather one of the lineages (Basilosauridae) survived in the form of mysticetes and odontocetes (similar to the evolution of dinosaurs, whereby Dinosauria per se did not go extinct but instead survived in the form of avian dinosaurs).

The detailed transitions from basilosaurids to mysticetes or odontocetes remain elusive due to the limited fossil materials available and the limitations of phylogenetic reconstructions, which only show sister-group relationships. A bracketing method similar to the well-developed Extant Phylogenetic Bracket approach (Witmer 1995) but using ontogenetic sequences (or an ontogenetic clade) to bracket a geologically older taxon may allow an ancestral species of the study taxon to be recognised (Tsai and Fordyce 2015a). While it remains challenging to determine whether fossil materials, which are often incomplete, belong to the same species or represent an ontogenetic sequence (Scannella and Horner 2010; Longrich and Field 2012), this bracketing approach offers a possible method for building up ancestor–descendant relationships to further dissect the stem-to-crown transition in the phylogenetic framework.

Eomysticetids have long been the focus of research considering the early transition from stem to crown mysticetes (Sanders and Barnes 2002a; Boessenecker and Fordyce 2015b; Coombs et al. 2022). However, Oligocene mysticetes from New Zealand, including *Toipahautea waitaki*, *Whakakai waipata*, *Horopeta umarere*, *Mauicetus parki*, and two undescribed specimens (OU 22224 and ZMT 67), have consistently been positioned phylogenetically more crownward of eomysticetids and sister lineages of crown mysticetes, as can be seen in the compilation of recent mysticete phylogenies (Tsai and Fordyce 2015b, 2018; Fordyce and Marx 2018; Shipps et al. 2019; Bisconti, Damarco, et al. 2021; Hernández-Cisneros and Nava-Sanchez 2022; Boessenecker et al. 2023) provided in a simplified form in Figure 1. The non-monophyletic and non-eomysticetid mysticetes then loosely form a *Toipahautea*-to-*Mauicetus* grade (in which *Toipahautea* is the oldest at ca. 27.5 Ma and *Mauicetus* is the youngest at ca. 24.7 Ma).

More interestingly, *Horopeta* and *Mauicetus* (and the undescribed specimens OU 22224 and ZMT 67) have also been phylogenetically nested within crown mysticetes (Figure 1), indicating their potential roles in bridging the mysticete stem-to-crown transition. For example, two synapomorphies (the well-developed crista transversa of the periotic and a horizontally-oriented tympanic sulcus of the bulla) have united *Horopeta umarere* within crown mysticetes, as a sister taxon of the cetotheriids (Tsai and Fordyce 2015b). Crown mysticetes include four clades (Balaenopteridae, Balaenidae, Eschrichtiidae, and Cetotheriidae) but, more broadly, can be separated into two major lineages (Balaenoidea and Balaenopteroidea + Cetotheriidae, including *Caperea marginata*; see Fordyce and Marx 2013). Of note, Bisconti et al. (2013) established the new superfamily Thalassotherii to include balaenopteroids and cetotheriids, the phylogenetic definition for which is USNM 187416 (undescribed specimen), *Balaenoptera*, their last common ancestor, and all the descendants of that ancestor (Bisconti et al. 2013), which did not include *Caperea* in the 2013 phylogenetic reconstruction. By contrast, the phylogenetic definition for Plicogulae provided by Geisler et al. (2011) included the most recent common ancestor of *Caperea marginata*, *Balaenoptera physalus*, and *Eschrichtius robustus*. The inclusion of different mysticetes in the phylogenetic definitions for Thalassotherii and Plicogulae seems to lead to contrasting interpretations. However, while the original definition for Thalassotherii, which was primarily based on fossil materials and morphological characters, did not include *Caperea*, the current consensus based on both molecular and morphological evidence supports the fact that *Caperea* is phylogenetically closer to balaenopteroids and cetotheriids than to balaenoids (e.g. Fordyce and Marx 2013; Wolf et al. 2023). Thus, it appears that Thalassotherii is essentially a synonym of Plicogulae. Phylogenetic uncertainties concerning Oligocene mysticetes from New Zealand (e.g. *Horopeta*, *Mauicetus*, and the undescribed specimens OU 22224 and ZMT 67) may reflect a rapid radiation event close to the origin of crown mysticetes, similar to the diversification burst that hinders our recognition of the evolution of early mammals (e.g. Goswami et al. 2022; Carlisle et al. 2023).

The existence of Oligocene balaenids remains uncertain due to the lack of a published description of OU 22224 (some phylogenetic studies have placed OU 22224 outside crown mysticetes; see Figure 1E or F). However, a brief descriptive account provided in a conference abstract (Fordyce 2002) indicates that OU 22224 shows some of the key diagnostic features for balaenids, including a prominently dorsoventrally arched skull and a lateromedially compressed rostrum, both of which contribute to the skim-feeding observed in this family. Modern morphological phylogenetic analyses tend to include as many characters as possible. However, interestingly, this approach may fail to recognise critical synapomorphies for lineages that radiated early from the most recent common ancestor. OU 22224 has been identified as a sister lineage of Balaenidae (Figure 1G; also see Tsai and Fordyce 2018), i.e. a stem balaenid, but this phylogenetic placement requires further testing until this specimen is formally described. If the interpretation that OU 22224 is an Oligocene balaenoid is correct, the origin of this balaenoid lineage can at least be traced back to the boundary of the Early and Late Oligocene (the dating of OU 22224 is ca. 27.3–26 Ma; see Marx and Fordyce 2015, supplementary information).

The other major mysticete lineage that leads to balaenopteroids and cetotheriids (collectively known as Pligogulae in Geisler et al. (2011) and Thalassotherii in Bisconti et al. (2013)) may be represented by the *Toipahautea*-to-*Mauicetus* grade. Unlike the balaenoid lineage that is likely represented by the undescribed OU 22224, each species in the *Toipahautea*-to-*Mauicetus* grade has been described and placed in various phylogenetic analyses (Benham 1937; Tsai and Fordyce 2015b, 2016, 2018), and *Horopeta* and *Mauicetus* have been recognised as crown mysticetes under various different analytical settings (Figure 1). However, species in the *Toipahautea*-to-*Mauicetus* grade are only known from the partially preserved type specimens. The better-preserved but still undescribed specimen OU 22545 (Fordyce 2005) may help to bridge the gap between Oligocene mysticetes and modern-looking balaenopteroids but, as with OU 22224, this hypothesis needs to be tested once OU 22545 has been fully described.

**Figure 2.**
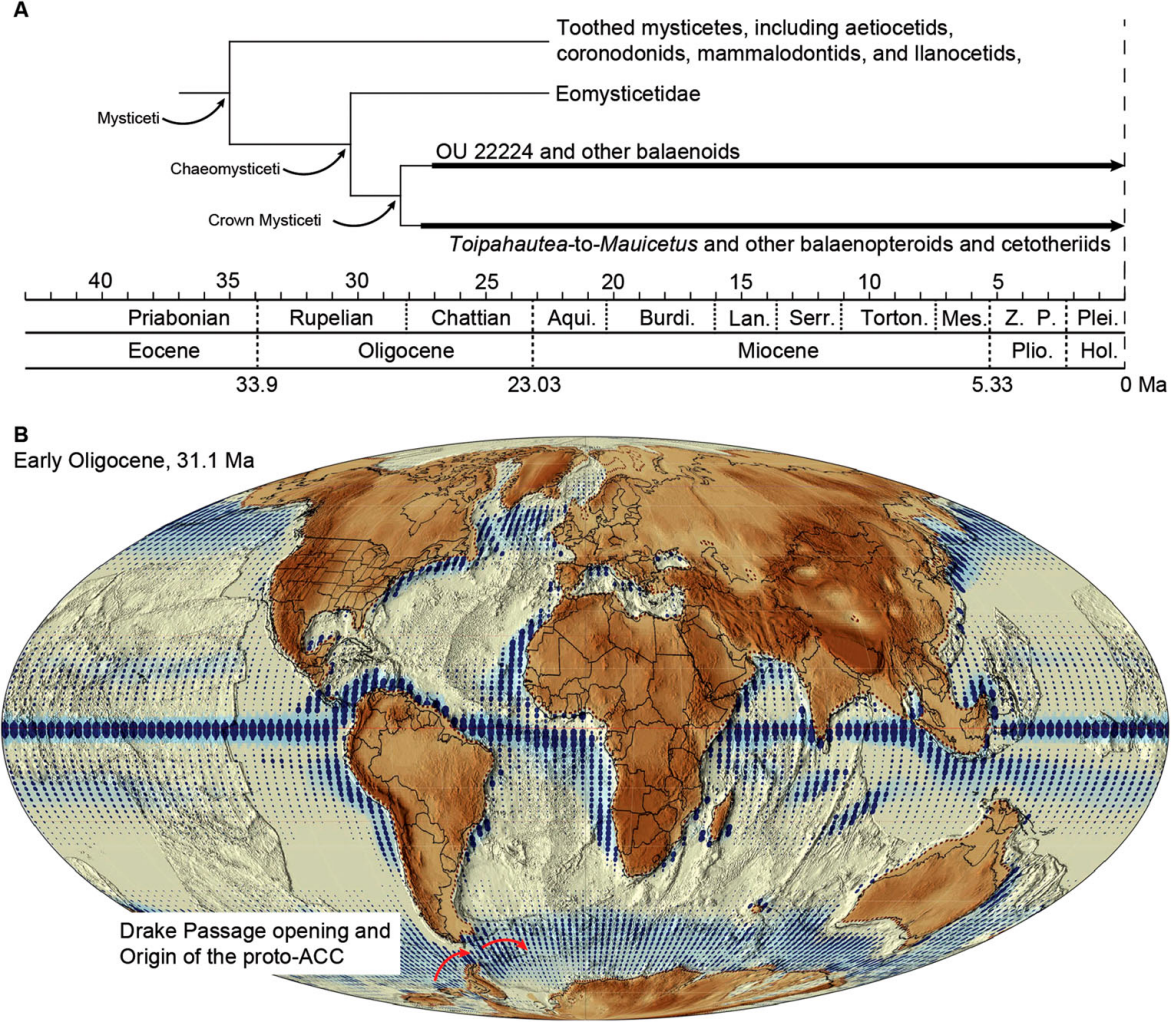
Phylogeny of the early mysticetes and origin of the possible environmental driver of their evolution, the Antarctic Circumpolar Current. **A**, Hypothetical phylogeny regarding the positions of non-eomysticetid mysticetes from the Oligocene deposits of New Zealand, including the *Toipahautea*-to-*Mauicetus* grade and the undescribed specimen OU 22224; **B**, world map from the Early Oligocene (ca. 31.1 Ma, reproduced from Scotese [2014] under the licence CC BY 4.0) with an indication of upwelling strength (blue shading) and the opening of the Drake Passage around the Eocene/Oligocene boundary (red arrow).

Given the possible positions of the *Toipahautea*-to-*Mauicetus* grade and the undescribed specimen OU 22224 close to or even within crown mysticetes, it appears that two major mysticete lineages may have appeared early in the Late Oligocene (more than 27 Ma) in New Zealand (Figure 2A), indicating that crown mysticetes likely originated at least as far back as the Early Oligocene (Rupelian) in the Southern Ocean. Early mysticete evolution has been broadly associated with the emergence of the Antarctic Circumpolar Current (ACC; Figure 2B); also see Fordyce 1977, 1980; Berger 2007; Steeman et al. 2009), which resulted in intense upwellings in the Southern Ocean and cascading ecological events (e.g. an abundance of nutrients, phytoplankton, zooplankton, and filter-feeding organisms). The ACC is the largest oceanic current system on Earth (Donohue et al. 2016) and affects three major ocean basins globally, but the precise timing of its onset remains debated (see Hodel et al. (2021) and references cited therein). It appears, however, that a proto-ACC likely already existed in the Early Oligocene, creating an environment that was ready for the upcoming origin and early radiation of crown mysticetes in the Southern Ocean (Figure 2). The ACC-mysticete hypothesis was first proposed almost half a century ago (Fordyce 1977), but its role in early baleen whale evolution remains largely uncertain. This updated synthesis (Figure 2), emphasising the importance of the *Toipahautea*-to-*Mauicetus* grade and the undescribed specimen OU 22224, attempts to lay a new foundation for future endeavours to further reveal the evolution of early crown mysticetes.

## Global radiation and functional evolution

**Figure 3.**
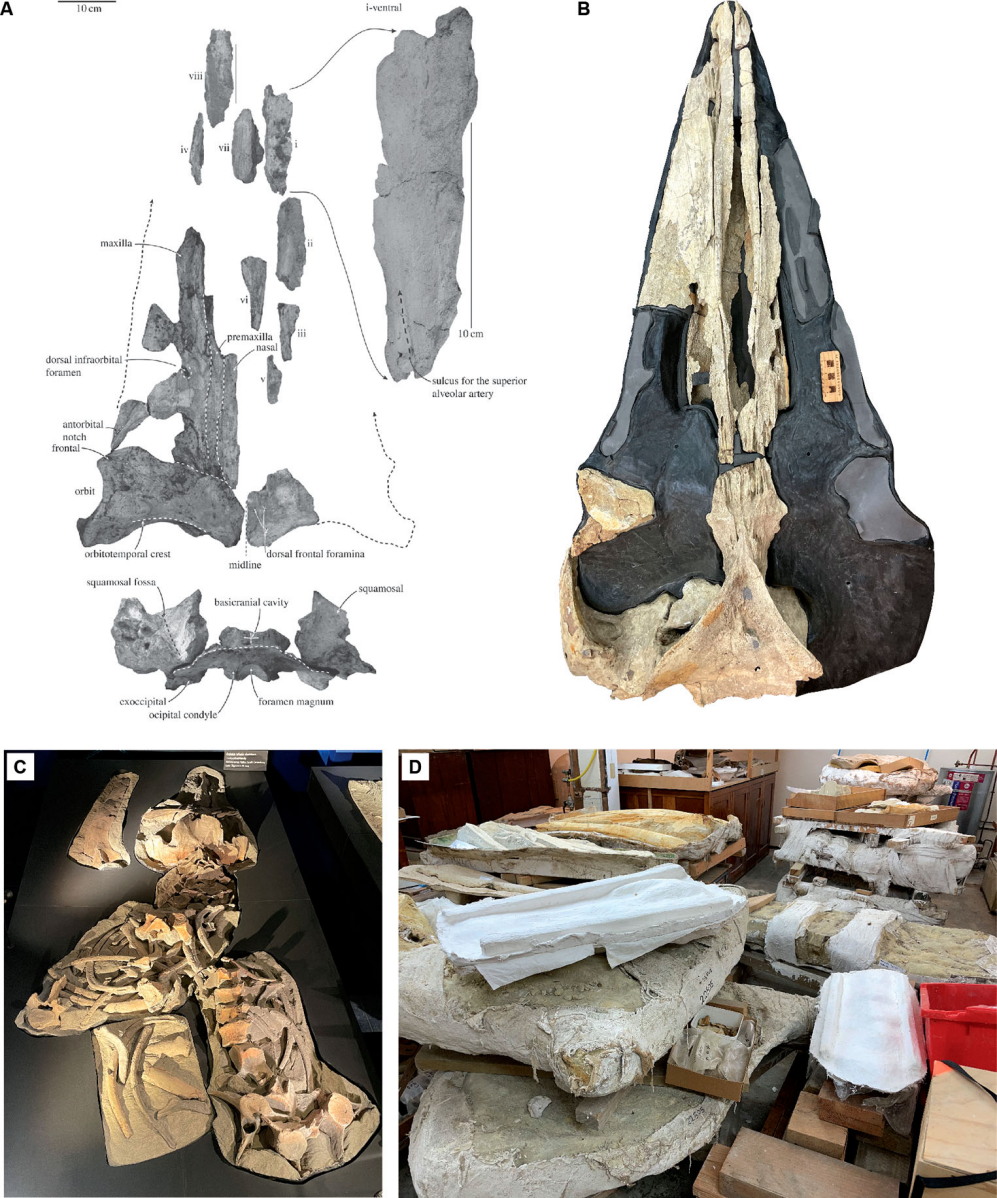
Selection of Oligocene mysticete specimens from the University of Otago’s Geology Museum, ranging from a published specimen to unprepared field jackets. **A**, *Toipahautea waitaki* OU 21981, representing the potential oldest crown mysticete known to date (reproduced from Tsai and Fordyce [2018] under the licence CC BY 4.0); **B**, OU 22545, a prepared but undescribed specimen; **C**, OU 21939, a partially prepared and undescribed specimen (currently on display at Tūhura Otago Museum); **D**, additional unprepared Oligocene mysticete specimens in the Geology Museum collection.

Aside from the *Toipahautea*-to-*Mauicetus* grade and abundant unprepared and undescribed Oligocene mysticete specimens from the Southern Ocean around New Zealand (Figure 3), the only Oligocene non-eomysticetid and non-toothed mysticetes found to date are *Sitsqwayk* (Peredo and Uhen 2016), *Maiabalaena* (Peredo et al. 2018), and *Tlaxcallicetus* (Hernández-Cisneros 2018) from the east coast of the North Pacific. Interestingly, a recent phylogenetic analysis (Boessenecker et al. 2023) showed that *Sitsqwayk* and *Maiabalaena* are both eomysticetids due to the new observations of seven eomysticetid synapomorphies, such as the presence of a secondary squamosal fossa and a ventral fossa on the apex of the zygomatic process. Consequently, both species are included in the Eomysticetidae in Figure 1A. The status of *Tlaxcallicetus* remains problematic, however, due to limited materials. In the original publication (Hernández-Cisneros 2018) and a subsequent analysis (Hernández-Cisneros and Nava-Sanchez 2022), *Tlaxcallicetus* showed a close relationship to *Sitsqwayk* (which is now considered an eomysticetid) (see Figure 1B). However, *Tlaxcallicetus* is one of the unresolved polytomies within the *Toipahautea*-to-*Mauicetus* grade (Boessenecker et al. 2023; Figure 1A), suggesting a possible early radiation of the *Toipahautea*-to-*Mauicetus* grade to the North Pacific. Consequently, more completely preserved specimens are required to elucidate the phylogenetic position of *Tlaxcallicetus* within Mysticeti.

Unlike eomysticetids, which have a global distribution (e.g. Boessenecker and Fordyce 2017a, 2017b), early possible crown mysticetes (*Toipahautea*, *Whakakai*, *Horopeta*, *Mauicetus*, and other undescribed specimens) appear to have a regional distribution in the Southern Ocean (only having been found in New Zealand to date). If no additional related Oligocene mysticetes are discovered in other regions, such as South America or the Northern Hemisphere, it may be that the *Toipahautea*-to-*Mauicetus* grade and other undescribed specimens (Figure 3) represent a genuine biological phenomenon, indicating the origin and early evolution of crown mysticetes in the vicinity of New Zealand, likely driven by the onset of the ACC (Figure 2). However, there are at least two other possible explanations for this observed distribution.

First, research on Oligocene mysticetes has largely focused on toothed mysticetes and eomysticetids, so it is possible that more effort in searching for early possible crown mysticetes will lead to relevant fossils being found outside New Zealand (e.g. the phylogenetic position of *Tlaxcallicetus* requires further research). *Toipahautea*, *Whakakai*, *Horopeta*, *Mauicetus*, and other undescribed specimens discovered to date represent abundant and relatively large mysticetes from the Oligocene deposits of New Zealand (Figure 3), and long-distance migration or dispersal is common in cetaceans (from Eocene archaeocetes [Lambert et al. 2019] to Pleistocene mysticetes [Tsai and Chang 2019]). Therefore, large species such as these are unlikely to have only inhabited New Zealand during the Oligocene but more likely experienced global radiation, a topic that requires further attention and research effort worldwide.

Second, eomysticetids may have already diversified and radiated globally in the Early Oligocene, occupying ecological niches that prevented later-diverging crown mysticetes from establishing populations across the globe. The occurrence of eomysticetids in the Early Oligocene (from 33.9–27.8 million years ago; Sanders and Barnes 2002b; Okazaki 2012) indicates that eomysticetids diverged from other early chaeomysticetes (one of which gave rise to crown mysticetes; Figure 2A) at least in the Early Oligocene. This scenario may not explain the coexistence of abundant eomysticetids and the *Toipahautea*-to-*Mauicetus* grade in the Oligocene deposits of New Zealand. However, extant baleen whales often avoid direct ecological competition through food partitioning – for example, blue whales predominantly devour euphausiids, while fin whales consume small fishes but also include euphausiids in their diet when they are superabundant (Würsig et al. 2018). Niche partitioning according to size has also been proposed for the Oligocene toothed mysticetes (Tsai and Ando 2016), so the coexisting eomysticetids and members of the *Toipahautea*-to-*Mauicetus* grade in the Oligocene of New Zealand may have exercised food partitioning using various baleen-assisted feeding strategies (e.g. *Horopeta* likely used an early form of gulp feeding, whereas eomysticetids employed primitive skim feeding), as well as other currently unknown mechanisms.

The defining features of a specific lineage often lie in the soft tissues (such as the keratinous baleen for Mysticeti and the echolocation-propagating melon for Odontoceti), but unfortunately soft tissues are rarely preserved in the fossil record. Thus, osteological correlates of these are required to allow interpretations of functionality and the underlying evolutionary implications. However, the identification of these is often problematic and highly debated. The possession of possible proto-baleen in toothed ‘baleen’ whales (Aetiocetidae) remains controversial (Ekdale and Deméré 2022; Peredo et al. 2022; also see recent publications on the early evolution of odontocete echolocation, including Geisler et al. (2014) and Racicot et al. (2019)). In addition to the characteristic baleen, a recently discovered sensory organ (Pyenson et al. 2012) may play a critical role in initiating filter-feeding behaviour in balaenopterids. although the recognition of osteological correlates to such a somewhat enigmatic sensory organ may require detailed anatomical work and creative experimental tests.

Computed tomography scanning technology on extant and fossil skulls and an improved understanding of functional linkages may offer novel clues to the antiquity or origin of certain soft tissues and the game-changing behaviours they are associated with. For example, the sternum (breastbone) is associated with the origin of the sternohyoideus muscle, which depresses and retracts the hyoid apparatus and thus is related to feeding. A recent study on the evolution of the mammalian sternum (Brent et al. 2023) may lay a new foundation for more in-depth research to understand the evolution of feeding in mysticetes based on the sternal structures. The bizarre rod-like sternum of *Horopeta umarere* and its robust connection to the ribs likely indicates the early phase of gulp/lunge feeding in early crown mysticetes. If the newly discovered sensory organ mentioned above facilitates gulp feeding in rorquals (Balaenopteridae) as suggested by Pyenson et al. (2012), the Oligocene *Horopeta* may indicate the timing of the origin and early evolution of this organ, which enables the feeding feast observed in extant rorquals.

## Concluding remarks

This review aims to attract attention to the less-studied topic of the origin and early evolution of crown mysticetes. The *Toipahautea*-to-*Mauicetus* grade and numerous undescribed fossil specimens in the collection at the University of Otago’s Geology Museum (such as OU 22224, a potential Oligocene right whale that remains undescribed) from the Oligocene deposits of New Zealand likely represent early crown mysticetes. However, for now, *Horopeta umarere* represents the only fully described species from these deposits that has been placed phylogenetically within crown mysticetes. The two synapomorphies that identify this species’ crown mysticete position close to cetotheriids are both found in the earbones (on the periotic and the tympanic bulla). Given that baleen and its associated feeding strategy play critical roles in the origin of crown mysticetes, future descriptions of the Otago collection and discoveries of more well-preserved skulls should offer further vital features for recognising the early evolution of crown mysticetes.

The stem-to-crown transition in mysticete evolution likely occurred in the Early Oligocene, and two major modern mysticete lineages show a possible early foundation around the boundary of the Early/Late Oligocene (ca. 28 Ma) rather than in the late Early Miocene (ca. 18 Ma) after a so-called ‘dark age’ (Marx et al. 2019). This review and future published descriptions of the undescribed specimens in the Otago collection will lay the foundations for an updated geological timeline that can be used by future researchers to estimate the divergence or early evolution of mysticetes, which will be particularly useful for those conducting molecular clock-based research (see Gatesy and McGowen [2021] for a recent summary of how molecular clock analyses have yielded disparate results).

Interestingly, the evolution of mysticetes may have been affected by not only drastic environmental change, such as the drop in sea level, but also a previously poorly known extinction event in the ocean during the Early Miocene (Sibert and Rubin 2021; but also see Feichtinger et al. 2021), which may have resulted in a large-scale ecological collapse. A Miocene gap appears to have reset the evolution of mysticetes and given rise to a possible radiation in crown mysticetes. However, the first episode of crown mysticete radiation likely occurred early in the Late Oligocene, and it is possible that crown mysticetes lived in the shadow of other early diversified stem mysticetes (such as toothed mysticetes and eomysticetids). Future research around the world and the formal descriptions of as yet undescribed fossils in the collection at the University of Otago’s Geology Museum should help us to better understand this poorly known chapter of crown mysticete evolution and further reveal the fall and rise of these giants.

## Institutional abbreviations

OU, Geology Museum, University of Otago, New Zealand; USNM, National Museum of Natural History, Smithsonian Institution, USA; ZMT, Canterbury Museum, New Zealand.
